# Ambulatory sentinel lymph node biopsy preceding neoadjuvant therapy in patients with operable breast cancer: a preliminary study

**DOI:** 10.1186/s12957-015-0471-3

**Published:** 2015-02-15

**Authors:** Shinichiro Kashiwagi, Naoyoshi Onoda, Yuka Asano, Kento Kurata, Satoru Noda, Hidemi Kawajiri, Tsutomu Takashima, Masahiko Ohsawa, Seiichi Kitagawa, Kosei Hirakawa

**Affiliations:** Department of Surgical Oncology, Osaka City University Graduate School of Medicine, 1-4-3 Asahi-machi, Abeno-ku, Osaka, 545-8585 Japan; Department of Diagnostic Pathology, Osaka City University Graduate School of Medicine, 1-4-3 Asahi-machi, Abeno-ku, Osaka, 545-8585 Japan; Department of Physiology, Osaka City University Graduate School of Medicine, 1-4-3 Asahi-machi, Abeno-ku, Osaka, 545-8585 Japan

**Keywords:** Breast cancer, Operation, Ambulatory surgical procedure, Sentinel lymph node biopsy, False negative

## Abstract

**Background:**

Sentinel lymph node biopsy (SNB)-oriented stepwise treatment under local anesthesia has been performed in the outpatient-ambulatory setting in patients receiving neoadjuvant therapy (NAT). We retrospectively reviewed our preliminary experience of ambulatory SNB in breast cancer patients scheduled to undergo NAT to evaluate the usefulness and feasibility of this method as a minimally invasive, stepwise treatment protocol.

**Methods:**

We retrospectively identified 56 patients with breast cancer without obvious nodal involvement who were scheduled to receive NAT before breast surgery. SNB was performed under local anesthesia in an ambulatory outpatient setting before the initiation of NAT.

**Results:**

The average number of removed sentinel lymph nodes was 1.9. Identification of the sentinel node was possible in all cases, and macrometastasis was observed in six cases (10.7%). Micrometastasis was observed in five cases, while isolated tumor cells were noted in six cases. There were no delays in the initiation of NAT as a result of complications of SNB.

**Conclusions:**

This pilot study demonstrated the safety and feasibility of ambulatory SNB prior to NAT. Further studies are warranted to assess the strict indications, patient satisfaction, and medical economics of this procedure.

## Background

Sentinel lymph node biopsy (SNB) in patients undergoing breast cancer surgery (BCS) is currently regarded as the standard minimally invasive strategy for managing clinically node-negative early breast cancer [1-5]. Neoadjuvant therapy (NAT) with chemo- or endocrine therapy is usually administered with the aim of enabling BCS or evaluating drug sensitivity *in situ* and is also regarded as a standard treatment for operable breast cancer [6,7]. Nodal status is a major factor determining the suitability of NAT. However, although SNB is considered to be an accurate method for evaluating axillary nodal status as an alternative to conservative axillary dissection, SNB after NAT is less reliable, particularly in patients in whom nodal involvement has been already detected prior to NAT [8,9]. Several reports have therefore suggested SNB prior to the initiation of NAT as a useful strategy for assessing axillary nodal status [10-13]. However, the application of SNB before NAT requires additional surgery and may result in delays in administering NAT. We have applied an ambulatory SNB protocol that allows NAT to be initiated based on an accurate histological diagnosis, including axillary nodal status, with minimal patient burden, thus enabling the immediate administration of NAT followed by BCS, with or without hospitalization, in accordance with the patient’s needs.

SNB can be performed safely and adequately under local anesthesia in the outpatient ambulatory setting, without hospitalization [14-20]. A pathological diagnosis can then be obtained from permanent preparations without the need for intraoperative evaluation of frozen sections, in which the latter process is associated with a 7% to 9% false-negative diagnosis rate [5,21-23]. Furthermore, axillary node dissection at surgery after NAT can be avoided in patients with pathologically proven sentinel lymph node (SN)-negative status [23-25].

In this study, we retrospectively reviewed our preliminary experience of ambulatory SNB in breast cancer patients scheduled to undergo NAT and evaluated the usefulness and feasibility of this method as an appropriate, minimally invasive, stepwise treatment protocol.

## Methods

### Patient characteristics

**Figure 1:**
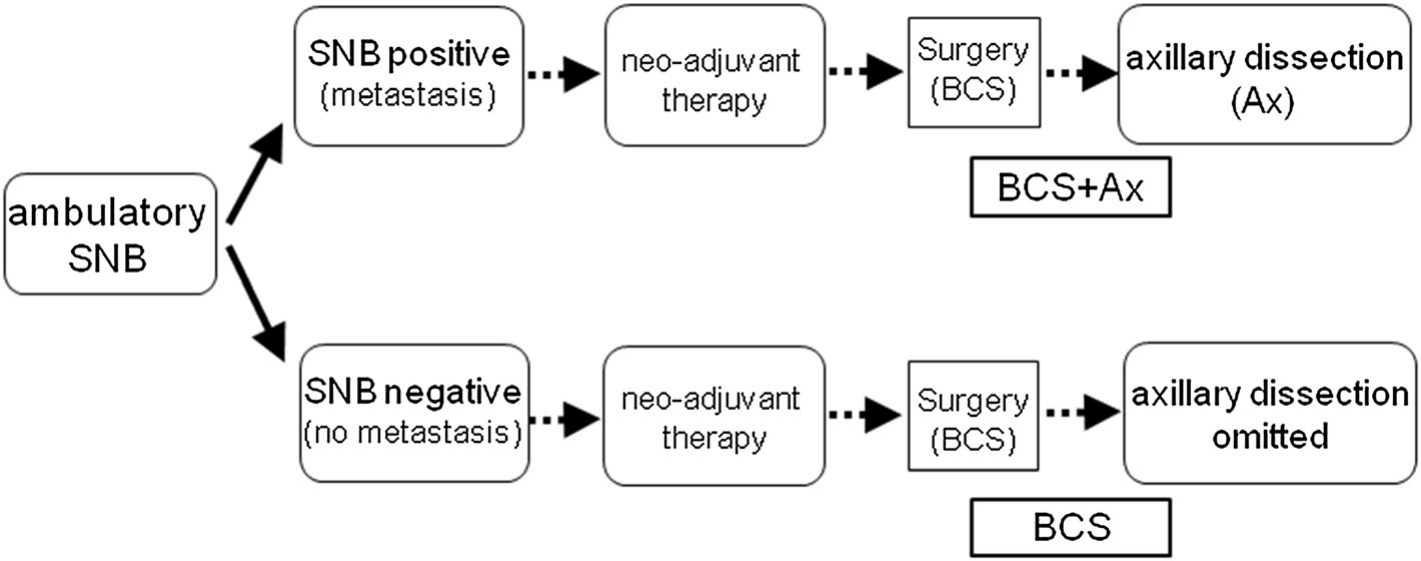
**Treatment plan.** We administered a series of treatments, including sentinel lymph node biopsy (SNB) under local anesthesia using ambulatory surgical procedures, followed by treatment with neoadjuvant therapy (NAT) based on the histological diagnosis, and subsequent breast cancer surgery (BCS). Ax: Axillary dissection.

This study included 56 patients with a histologically confirmed diagnosis of invasive breast cancer following core needle biopsy (CNB) of the tumor between April 2009 and August 2013. All patients were scheduled for BCS following the administration of NAT. Patients with clinical T2 tumors in whom the absence of distant metastasis (M0) was confirmed by computed tomography (CT) and chest and abdomen and bone scintigraphy were enrolled. Patients with multiple tumors or with a previous history of surgery to the affected breast were excluded. Axillary nodal status was evaluated by palpation, CT, and ultrasonography, and patients with obvious node metastasis were also excluded to avoid unnecessary SNB. In these patients, lymph node metastasis was confirmed by either fine needle aspiration cytology or CNB of the affected nodes. Patients fulfilling the inclusion criteria were provided with sufficient explanation of the stepwise treatment plan (Figure 1), including the procedure for ambulatory SNB with local anesthesia.

This study was performed in accordance with the Declaration of Helsinki and carried out with the approval of the Ethical Review Board of Osaka City University (#926). Sufficient explanation was provided and written informed consent was obtained from all study subjects for their involvement in this study and for the storage and use of their data.

### Surgical procedure

**Figure 2:**
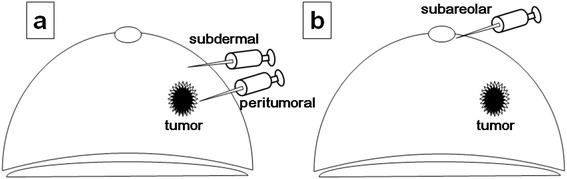
**Sentinel node examination. (a)** Radioisotope examination: peritumoral injection of 99mTc-phytate 1 day before surgery. **(b)** Dye examination: intradermal injection of indocyanine green in the areola.

SNs were identified by a combination of radioisotope and dye methods [26]. For radioisotope examination, intradermal and subcutaneous 99mTc-phytic acid colloid 1 mCi (1 mL) was injected into the skin overlying the tumor and in the vicinity of the tumor the day before ambulatory SNB. Lymphoscintigraphy was performed 4 h after injection (Figure 2a). For dye examination, 1.0 mL of 1% lidocaine (not containing epinephrine) was added to 4.0 mL of indocyanine green (5 mg/mL) solution and injected intracutaneously in the areola of the affected side (Figure 2b). Approximately 10 min after injection, a skin incision (approximately 2 cm) was made in the axilla under local anesthesia with 0.5% to 1.0% lidocaine (containing 1:100,000 epinephrine) to identify the blue node [14]. A hand-held gamma probe was used to confirm the accumulation of radioactivity in the SN injected the day before SNB. No drainage tubes were placed. Pathological diagnosis of lymph node metastasis was made by slicing the entire SN into 2-mm-thick sections, and a detailed pathological diagnosis was obtained after conventional hematoxylin-eosin staining associated with touch imprint cytology [27,28], by a pathologist specializing in breast cancer. A positive diagnosis of SN metastasis as an indication for axillary clearance was defined as macrometastasis (i.e., tumor diameter >2 mm) in the SN. Micrometastasis (i.e., tumor diameter >0.2 mm, ≤2 mm, or <200 tumor cells) and isolated tumor cells (ITC, i.e., tumor diameter <0.2 mm or <200 tumor cells) were determined as negative indications [29].

NAT was generally recommended according to the intrinsic subtype of the primary tumor determined from the CNB sample, and axillary dissection was followed by BCS under general anesthesia within 4 weeks after the termination of NAT in SN-positive patients. BCS without axillary dissection was applied in SN-negative patients with or without hospitalization, according to patient preference. Subsequent radiotherapy with 50 to 60 Gy was administered to the residual mammary gland. Standard adjuvant therapy was administered if indicated, according to tumor subtype.

## Results

All patients were female, with a median age of 59 (range 28 to 76) years. All SNB procedures were accomplished under local anesthesia without conversion to general anesthesia. No patients required hospitalization after SNB. There were no complications of ambulatory SNB requiring treatment, such as bleeding, infection, or lymphorrhea. The average number of excised SNs was 1.9 ± 1.1 (Table 1), and SN identification was possible in all cases.

**Table 1:** **Demographical data for 56 patients with ambulatory sentinel lymph node biopsy undergoing breast cancer treatment**

**Parameters (** ***n*** **= 56)**	**Results**
Age (years old)	59 ± 12
The average number of extracted SN	1.9 ± 1.1
Tumor location right/left	25 (44.6%)/31 (55.4%)

*SN* sentinel lymph node.

Macrometastasis was detected in nine SNs in six patients (10.7%) (median diameter of metastatic lesion: 7.00 ± 2.20 mm). Micrometastasis was observed in ten SNs in five patients (median diameter: 0.60 ± 0.23 mm), and ITC was observed in ten SNs in six patients (median length: 0.14 ± 0.06 mm) (Table 2). NAT was performed according to each intrinsic subtype in all 56 patients after evaluating the histological status of the axillary lymph node metastasis. Neoadjuvant chemotherapy (NAC) was conducted in 50 cases, resulting in 16 pathological complete responses (pCRs), 29 pathological partial responses (pPRs), and 5 cases of pathological stable disease (pSD). No cases of pathological progressive disease (pPD) were observed. Endocrine therapy was administered in six cases, resulting in no pCRs or pPDs, two cases of pPR, and four cases of pSD. BCS with axillary lymph node dissection was subsequently carried out as the second-phase surgical treatment in six SNB-positive patients, and further, axillary lymph node involvement was found in two of these six patients. Three additional nodes were found to be involved during axillary lymph node dissection after NAT in one patient with a single positive SN before NAT. Two additional metastatic nodes were found after NAT in another patient with three positive SNs before NAT. No additional axillary lymph node metastases were found in the other four patients who underwent axillary lymph node dissection (Table 3). BCS without axillary lymph node dissection was performed in the remaining 50 patients, including 7 patients under local anesthesia and 43 patients under general anesthesia. All patients remained alive without disease after a median follow-up period of 28 (range 3 to 52) months since BCS.

**Table 2:** **Results of sentinel lymph node biopsy**

	**Number of cases**	**Number of SN**	**Size of metastatic lesion (mm)**
Metastasis negative			
No metastasis	39	79	
ITC	6	10	0.14 ± 0.06
Micrometastasis	5	10	0.60 ± 0.23
Metastasis positive			
Macrometastasis	6	7	7.00 ± 2.20
Total	56	106	

*SN* sentinel lymph node. Macrometastasis (tumor diameter >2 mm); micrometastasis (tumor diameter >0.2 mm, ≤2 mm, or <200 tumor cells); ITC (isolated tumor cell; tumor diameter <0.2 mm or <200 tumor cells).

**Table 3:** **Results of axillary node dissection**

**Patient #**	**Sentinel node biopsy**		**Axillar dissection**	
	**Number of SN sampled**	**Number of SN with metastasis**	**Number of node sampled**	**Number of node with metastasis**
1	1	1	12	3
2	1	1	12	0
3	1	1	9	0
4	2	1	8	0
5	2	2	8	0
6	3	1	13	2
Total	10	7	62	5

*SN* sentinel lymph node.

## Discussion

The long-term prognosis of SNB patients is not affected by omitting axillary lymph node dissection, according to the results of the large-scale clinical trial NSABP B-32 [23]. SNB-oriented management of axillary lymph node dissection has thus become the standard, minimally invasive surgical strategy for controlling local disease and evaluating the pathological stage in patients with clinically node-negative, early breast cancer [1-5]. General anesthesia is contraindicated in some patients because of co-morbidities or a desire for ambulatory or short-stay surgery. We therefore developed an ambulatory SNB-oriented stepwise treatment strategy for such patients, as described in this study. Surgical methods for performing axillary lymph node dissection under local anesthesia have been reported previously, but the procedure can result in insufficient dissection because of unsatisfactory analgesia [19,20]. General anesthesia is therefore necessary for standard axillary lymph node dissection. SNB, however, can be carried out adequately and safely under local anesthesia, as described in the present study [14-18]. The existence and number of lymph node metastases are major prognostic factors [30,31], and obtaining an accurate diagnosis is mandatory for selecting patients who require additional systemic therapy after surgery. The false-negative rate of SNB for breast cancer is reported to be 7% to 9%, based on the results of meta-analyses [5,21-23]. The major reason for failure with respect to pathological misdiagnosis is thought to involve inadequate intraoperative tissue diagnosis associated with the limitations of tissue-preparation conditions during surgery using frozen specimens. Accordingly, non-definitive results have been demonstrated in 3% to 35% of cases [27,32-35]. These false-negative or indeterminate results may be avoided by standard pathological investigations using formalin-fixed paraffin-embedded samples obtained by ambulatory SNB under local anesthesia.

The administration of NAT may affect the accuracy of SNB. Two large-scale studies demonstrated that performing SNB after NAC was not recommended in patients with apparent axillary lymph node involvement before treatment because of the lower detection and higher false-negative rates (SENTINA trial 14.2%, ACOSOG Z1071 trial 12.6%) of SNB after NAC [36,37]. At the same time, the authors suggested that patients without nodal disease may be candidates for SNB after NAC to avoid repeated surgery, delays in the initiation of NAC, and possible unnecessary axillary dissection in cases where total eradication of nodal disease has been achieved by NAC. Although controversies remain, SNB prior to NAT represents the most reliable way to evaluate axillary lymph node metastasis in patients scheduled for NAT. This procedure could help to predict the effect of NAT on the involved axillary lymph nodes by confirming pathological nodal involvement before NAT. Ambulatory SNB may also minimize the burden on the patient with respect to repeated surgery and delays in administering NAT, as demonstrated in the present study. Furthermore, intimate pathological review of the involved node may aid precise stratification of the patients for additional therapy and follow-up protocols after scheduled NAT and BCS. The current study did not evaluate the appropriate indications for SNB in patients in whom NAT failed, and this issue remains a concern. Patients demonstrating resistance to NAT may require changes in their treatment strategy from chemotherapy to surgical therapy to achieve sufficient disease control. Among such cases, even in those with previous negative results on SNB, simultaneous axillary lymph node dissection may be necessary to confirm negative node involvement and establish complete local control by salvage surgery.

According to the results of the recent Z0011 trial [38], clinicians must reconsider the need for complete axillary dissection in SNB-positive patients with clinically node-negative, early breast cancer scheduled for BCS with radiation and adjuvant therapy, as demonstrated in the present study. Furthermore, the present results also indicate the possibility of skipping SNB in such cases, though further investigations with longer observation periods are required. However, our SNB-oriented stepwise treatment protocol is likely to be beneficial in patients with clinically node-negative, early breast cancer, by offering a sufficient but minimally invasive treatment strategy based on accurate pathological staging.

## Conclusions

This pilot study demonstrated the safety and feasibility of ambulatory SNB prior to NAT in patients with operable breast cancer. Future studies, including the determination of strict indications, assessments of patient satisfaction, and medical economics, are warranted.

## Abbreviations

BCS: breast cancer surgery
CNB: core needle biopsy
CT: computed tomography
ITC: isolated tumor cell
NAT: neoadjuvant therapy
pCR: pathological complete response
pPD: pathological progressive disease
pPR: pathological partial response
pSD: pathological stable disease
SN: sentinel lymph node
SNB: sentinel lymph node biopsy
